# Leishmaniasis: Bone Marrow Aspirate Smear and Rapid Antibody Test

**DOI:** 10.4274/tjh.2017.0291

**Published:** 2017-12-01

**Authors:** Beuy Joob, Viroj Wiwanitkit

**Affiliations:** 1 Sanitation 1 Medical Academic Center, Bangkok, Thailand; 2 DY Patil University Faculty of Medicine, Pune, India

**Keywords:** Non-Hodgkin lymphoma, miR-155, Prognostic value

## To The Editor,

The publication by Dorji et al. [1], “Microscopic Image of Leishman-Donovan Bodies in Bone Marrow Aspirate Smear of Patient Suffering from Unexplained Intermittent Low-Grade Fever and Cough”, is very interesting. Indeed, the clinical presentation of leishmaniasis is usually nonspecific and it is hard to discriminate it from other tropical infections [2]. However, the common presentation that might be useful for the inclusion of leishmaniasis in differential diagnosis is splenomegaly [2]. In the present report, an important observation is a negative antibody test for Leishmania pathogens. The gold standard for the diagnosis of leishmaniasis is usually the examination of the blood or marrow and identification of the parasite. However, it is considered harmful. A new rapid test might be a solution and a less invasive technique. Nevertheless, the problem of the diagnostic properties of the available rapid test is frequently reported [3]. There are many possible explanations for false negative results. One important explanation is the prozone phenomenon [4] due to heavy infection. In the present case, there might be parasites and excessive antigens that could induce false negative results due to the immunological testing and the dilution of the blood sample before the test could be a simple method to reduce the problem of the prozone effect. Focusing on the examination of bone marrow a spirates, a similar problem with diagnostic false negatives can be expected [5]. At present, the test with the best diagnostic properties is the polymerase chain reaction-based test [5].
